# Aufklärungserfolg, Zufriedenheit und Verbesserungsmöglichkeiten bei Computertomographie-Aufklärungen: Eine Befragung von Patienten und Ärzten

**DOI:** 10.1007/s00117-020-00727-w

**Published:** 2020-07-29

**Authors:** D. Vogele, O. Schöffski, K. Efinger, S. A. Schmidt, M. Beer, D. Kildal

**Affiliations:** 1 Klinik für Diagnostische und Interventionelle Radiologie, Universitätsklinikum Ulm, Albert-Einstein-Allee 23, 89081 Ulm, Deutschland; 2 Lehrstuhl für Gesundheitsmanagement, Friedrich-Alexander-Universität Erlangen-Nürnberg, Nürnberg, Deutschland; 3 Bundeswehrkrankenhaus Ulm, Ulm, Deutschland

**Keywords:** Aufklärung, Inhalt, Patientenkenntnisse, Technische Innovationen, Digitale Aufklärung, Informed consent, Content, Patient knowledge, Technical innovations, Electronic patient consent

## Abstract

**Hintergrund:**

Die Aufklärung eines Patienten vor einer Computertomographie (CT) spielt sowohl für die aufklärenden Ärzte als auch für die Patienten eine entscheidende Rolle. Ein persönliches Aufklärungsgespräch über die Durchführung, Risiken und mögliche Alternativen ist vor einer CT-Untersuchung verpflichtend.

**Methode:**

Durchgeführt wurde eine Befragung zur Patientenzufriedenheit hinsichtlich der Dauer und den Inhalten einer CT-Aufklärung. Befragt wurden hierüber auch aufklärende Ärzte. Ein weiterer Teil der Befragung beschäftigte sich mit der Akzeptanz technischer Hilfsmittel, wie z. B. Informationsvideos oder Tablets/PCs.

**Ergebnis:**

Insgesamt 512 Patienten und 106 Ärzte beteiligten sich an der Befragung. Die Dauer des Aufklärungsgesprächs gaben die Patienten mit durchschnittlich 4,08 min und die Ärzte mit 4,7 min an. Am ausführlichsten klärten die Ärzte über die Nebenwirkungen von Kontrastmitteln auf. Über mögliche Alternativen und die Notwendigkeit der Untersuchung wurde weniger aufgeklärt. Korrelierend erinnerten sich rund 92 % aller Patienten nicht an eine Information über alternative Untersuchungsmöglichkeiten. 88,7 % der Patienten und 95,3 % der ärztlichen Teilnehmer befürworteten die Aufklärung mithilfe von interaktiven Videos und Animationen und 74 % der Patienten sowie 98,8 % der Ärzte die Beantwortung der Fragen zum Gesundheitszustand am Tablet/PC.

**Schlussfolgerung:**

Die Dauer einer CT-Aufklärung wurde von den Patienten etwas kürzer eingeschätzt, wobei sich die Patienten teilweise nur schlecht an die Aufklärungsinhalte erinnerten. Die Akzeptanz gegenüber technischen Neuerungen war bei den Teilnehmern sehr hoch. Durch den Einsatz von Informationsvideos und Tablets/PCs könnte der Aufklärungserfolg erhöht werden.

## Abstract (en)

**Background:**

Providing informed consent before a computed tomography (CT) is important for the physicians and the patients. A personal interview about the procedure, risks, and possible alternatives is mandatory before a CT examination.

**Methods:**

A survey was carried out on patient satisfaction with regard to the duration and content of informed consents of CT examinations. Physicians were also interviewed about the duration and content of CT informed consents. Another part of the survey dealt with the acceptance of technical innovations, such as information videos or tablets/PCs.

**Results:**

A total of 512 patients and 106 physicians took part in the survey. The duration of the informed consent was estimated by the patients to be 4.08 min on average and 4.7 min by the physicians. The most detailed information given by the physicians regards side effects associated with contrast agents. Less information was given on possible diagnostic alternatives and the need for an examination. According to this, about 92% of all patients did not remember having received information about alternative examinations. Furthermore, 88.7% of the patients and 95.3% of the physicians recommended informed consent using interactive videos and animations, and 74% of the patients and 98.8% of the physicians recommended answering questions on tablet/PC.

**Conclusion:**

Patients estimated the duration of a CT informed consent to be a little bit shorter, although some patients did not remember the content very well. The acceptance of technical innovations was very high among the participants. The use of information videos and tablets/PCs could increase the success of providing informed consent.

Der Aufklärung eines Patienten vor einer medizinischen Maßnahme wie der Computertomographie (CT) kommt eine entscheidende Bedeutung zu. Die durchführenden Ärzte sind verpflichtet, vor der Untersuchung ein Aufklärungsgespräch durchzuführen und dieses zu dokumentieren [1]. Das Delegieren an nichtärztliches Personal ist nicht zulässig [27, 28].

Bei elektiven Untersuchungen muss die Aufklärung ausführlich erfolgen und so rechtzeitig vor der Behandlung, „dass der Patient seine Entscheidung über die Einwilligung überlegt treffen kann“ [2]. Für die durchführenden Ärzte stellen die Aufklärungsgespräche vor einer CT häufig eine zeitliche Belastung dar. Hinzu kommt noch die Zeit für die verpflichtende umfassende Dokumentation des Aufklärungsgesprächs [4, 16].

Das ärztliche Aufklärungsgespräch muss für die Patienten verständlich sein [2]. Dies ist nicht zuletzt im Hinblick auf die teilweise erheblichen Unterschiede bezüglich der Gesundheitskompetenz verschiedener Patienten eine große Herausforderung. Die Gesundheitskompetenz wird entscheidend durch demographische, psychosoziale und kulturelle Faktoren beeinflusst [26]. Neben dem Alter, dem Geschlecht und dem kulturellen Hintergrund haben auch Fähigkeiten wie Sehkraft, Hörvermögen, sprachliche Fertigkeiten, Gedächtnisleistung und logisches Denken einen entscheidenden Einfluss [13, 15].

Als Unterstützung für ein Aufklärungsgespräch werden häufig Informationsbögen verwendet, die auch im Falle radiologischer Maßnahmen die entsprechenden Informationen enthalten. Die Informationsbögen dürfen nach § 630e Absatz 2 BGB ergänzend verwendet werden [2]. Ein Aufklärungsgespräch können sie jedoch nicht ersetzen. Dem Patienten sind Abschriften von Unterlagen, die er im Zusammenhang mit der Aufklärung oder Einwilligung unterzeichnet hat, auszuhändigen [25]. Auch eine Kopie des Aufklärungsbogens muss dem Patienten nach der Aufklärung, jedoch vor der Untersuchung ausgehändigt werden.

In der vorliegenden Studie sollte die aktuelle Situation hinsichtlich der Zufriedenheit ärztlicher Aufklärungen für CT-Untersuchungen aus Sicht der Ärzte und der Patienten evaluiert werden. Die Befragung unter den Patienten beleuchtet die aktuelle Aufklärungssituation einschließlich der Ergebnisqualität. Hinsichtlich der Ärzte sollte zudem analysiert werden, wie die Durchführung und der Inhalt der Aufklärungsgespräche im Alltag wahrgenommen werden. Außerdem wurde die Akzeptanz technischer Innovationen im Rahmen eines Aufklärungsgesprächs wie Tablet/PC oder Video-Aufklärungen abgefragt.

## Methode

### Befragung zur Patientenzufriedenheit bei der CT-Aufklärung

Zur Teilnahme wurden die Mitglieder von 116 Selbsthilfegruppen für verschiedene Krankheitsbilder auf Bundes- und Länderebene eingeladen. Außerdem erfolgte die Bekanntgabe der Befragung über sog. „Schwarze Bretter“ im Internet in 21 Städten in Deutschland. Zusätzlich wurden Fragebögen in zwei Kliniken der Maximalversorgung an Patienten ausgegeben, die dort im Zeitraum Juli bis September 2016 eine CT mit Kontrastmittel erhielten.

Die Teilnehmer wurden neben der Angabe des Alters gebeten, abzuschätzen, wie lange die Aufklärung dauerte, und anzugeben, ob sie eine Aufklärung in Zukunft kürzer, länger oder in gleicher Länge bevorzugen würden. Die Teilnehmer wurden gefragt, an welche Bestandteile der Aufklärung sie sich erinnerten. Im nächsten Schritt wurde gefragt, wie gut sich die Patienten hinsichtlich des Ablaufs, der Notwendigkeit der Untersuchung bzw. möglicher diagnostischer Alternativen sowie hinsichtlich der Risiken der CT und den Nebenwirkungen der eingesetzten Kontrastmittel informiert fühlten. Abschließend wurden die Teilnehmer gefragt, ob sie sich in Zukunft vorstellen können, mithilfe von Videos und Animationen am Tablet/PC aufgeklärt zu werden.

### Befragung unter Ärzten zu Aufwand und Verbesserungsmöglichkeit bei der CT-Aufklärung

Die Befragung wurde per Inserat den Mitgliedern des Ärztenachrichtendienstes AEND.de und der Internetseite Medilearn.de bekannt gemacht. Außerdem wurden Ärzte in radiologischen Abteilungen mehrerer großer Kliniken, 5 Universitätskliniken und insgesamt 30 Großpraxen für Radiologie per E‑Mail zur Teilnahme an der Befragung eingeladen. Die Befragung lief im Zeitraum August bis Oktober 2016 über LimeSurvey.

Eruiert wurde die durchschnittliche Anzahl der durchgeführten CT-Aufklärungen pro Tag mit den Auswahlkategorien weniger als 5, 5–10, 10–15 oder mehr als 15 Aufklärungen. Im Anschluss wurde gefragt, ob in der Einrichtung bereits moderne Medien zur Aufklärung genutzt werden. Ergänzend wurde nach der geschätzten Dauer einer Aufklärung gefragt.

Des Weiteren wurden die Teilnehmer gebeten, anzugeben, wie ausführlich sie hinsichtlich des Ablaufs und Dauer der Untersuchung, Notwendigkeit der Untersuchung, mögliche Alternativen, Risiken der Untersuchung und Nebenwirkungen verwendeter Kontrastmittel aufklären. Anschließend wurde gefragt, ob Aufklärungen die Abläufe der Teilnehmer in ihrem Arbeitsalltag beeinträchtigen. Die Teilnehmer sollten die Compliance der Patienten einschätzen und angeben, zu wie viel Prozent die Patienten die Aufklärungsbögen und Gesundheitsfragen nach ihrer Meinung lesen und verstehen konnten. Der letzte Teil der Befragung beschäftigte sich mit der Akzeptanz technischer Neuerungen.

## Ergebnisse

### Befragung zur Patientenzufriedenheit bei der CT-Aufklärung

Insgesamt 512 Teilnehmer beteiligten sich an der Befragung, davon über die Online-Befragung 361 und in den Kliniken 151. Da nicht alle Teilnehmer alle Fragen beantwortet haben, wird im Folgenden stets die Anzahl der abgegebenen Antworten zu der Frage angegeben. Prozentuale Angaben beziehen sich auf die Anzahl der abgegebenen Antworten zu der entsprechenden Frage.

Elf Teilnehmer hatten ihr Alter nicht angegeben. Von den übrigen 501 Teilnehmern waren 7 jünger als 18 Jahre (1,4 %), 93 zwischen 18 und 30 Jahren (18,5 %), 79 zwischen 31 und 40 Jahren (15,8 %), 89 zwischen 41 und 50 Jahren (17,8 %), 115 zwischen 51 und 60 Jahren (23 %), 67 zwischen 61 und 70 Jahren (13,3 %) und 51 über 70 Jahre (10,2 %).

Eine Einschätzung der Dauer der Aufklärung wurde von 395 Teilnehmern angegeben (Tab. 1). Der Wunsch nach einem künftig längeren, kürzeren oder gleich langen Aufklärungsgespräch wurde nicht von allen dieser Teilnehmer beantwortet. Bei einer Dauer unter 3 min wünschten die meisten Patienten eine längere Aufklärung (48 %). Die Zufriedenheit stieg mit der Dauer der Aufklärung und war in der Gruppe von 5–10 min am höchsten. Zu der Frage, an welche Bestandteile der Aufklärung sich die Teilnehmer erinnern, machten 343 Teilnehmer Angaben (Tab. 2).

**Table 1:** 

Geschätzte Dauer des Aufklärungsgesprächs (min)	*n* = 395 (%)	Anzahl der Teilnehmer, die ein längeres Gespräch wünschen (% der Untergruppe)	Anzahl der Teilnehmer, die ein ähnlich langes Gespräch wünschen (% der Untergruppe)	Anzahl der Teilnehmer, die ein kürzeres Gespräch wünschen (% der Untergruppe)
<3	125 (31,6 %)	60 (48 %)	53 (42,4 %)	12 (9,6 %)
3–5	151 (38,2 %)	20 (13,2 %)	103 (68,2 %)	20 (13,2 %)
5–10	97 (24,6 %)	7 (7,2 %)	77 (79,4 %)	10 (10,3 %)
>10	22 (5,6 %)	2 (9 %)	13 (59 %)	5 (22,7 %)

**Table 2:** 

*An welche Bestandteile der Aufklärung erinnern sich die Teilnehmer*	*Alle Teilnehmer mit einer oder mehreren Angaben (n* *=* *343)*	*Online-Teilnehmer mit einer oder mehreren Angaben (n* *=* *234)*	*Klinik-Teilnehmer mit einer oder mehreren Angaben (n* *=* *109)*
Strahlenbelastung/Strahlenrisiko	213 (62 %)	142 (60,7 %)	71 (65,1 %)
Alternative Untersuchungsmöglichkeiten	28 (8,2 %)	19 (8,1 %)	9 (8,3 %)
	*Teilnehmer mit KM-Untersuchung (% von 300)*	*Online-Teilnehmer mit KM-Untersuchung (% von 191)*	*Klinik-Teilnehmer mit KM-Untersuchung (% von 109)*
Allergien gegen eingesetzte Kontrastmittel	199 (66,3 %)	122 (61,3 %)	77 (70,6 %)
Schilddrüsenstörungen durch Kontrastmittel	113 (37,7 %)	49 (25,6 %)	64 (58,7 %)
Nierenfunktionsstörung durch Kontrastmittel	156 (52 %)	77 (40,3 %)	79 (72,5 %)

*KM* Kontrastmittel

**Figure 1:**
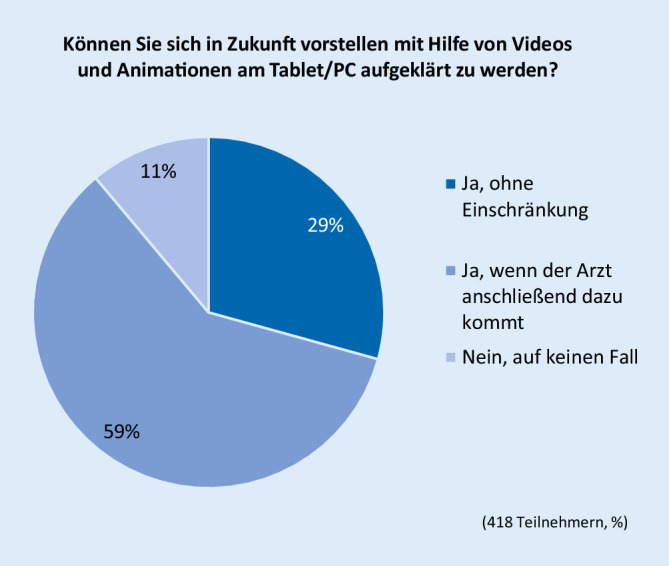

Die Frage, ob in Zukunft eine Aufklärung mithilfe von Videos und Animationen am Tablet/PC vorstellbar wäre, haben 418 (81,6 %) von 512 Teilnehmern beantwortet (Abb. 1). Insgesamt 371 (88,7 %) Teilnehmer haben die Frage mit „Ja“ beantwortet. Nur 47 (11,2 %) Teilnehmer lehnten die Aufklärung mit Videos und Animationen am PC grundsätzlich ab. 296 Teilnehmer (74 %) würden die Gesundheitsfragen am Tablet/PC beantworten.

### Befragung unter Ärzten zu Aufwand und Verbesserungsmöglichkeit bei der Aufklärung für CT-Untersuchungen

Insgesamt 106 Ärzte beteiligten sich an der Befragung, davon 81 radiologisch tätige Ärzte (46 Assistenzärzte, 14 Fachärzte, 17 Oberärzte und 4 Chefärzte) sowie 18 Ärzte, die in anderen Fachrichtungen tätig sind. Bei 7 Teilnehmern fehlte die Angabe der Fachrichtung. Nicht alle Teilnehmer haben alle Fragen beantwortet, daher werden im Folgenden bei Prozentzahlen immer die Anzahl der abgegebenen Antworten der betreffenden Frage zugrunde gelegt.

Die Frage, wie viele Aufklärungen (CT mit und ohne Kontrastmittel) der Teilnehmer im Tagesdurchschnitt durchführt, beantworteten 94 von 106 Teilnehmern. Bei den Oberärzten und Chefärzten der Radiologie klären 58 % bzw. 75 % weniger als 5 Patenten pro Tag über eine CT-Untersuchung auf. Dagegen geben 91 % der Assistenzärzte an, mehr als 5 Patienten pro Tag über eine CT-Untersuchung aufzuklären, 46 % sogar mehr als 10.

Von 94 Teilnehmern, die die Frage nach verwendeten Hilfsmitteln beantworteten, hatten 23 (24,5 %) einen eigenen Aufklärungsbogen, 73 (77,6 %) verwendeten einen kommerziell erworbenen Aufklärungsbogen. PC- oder Tablet-gestützte Anamnesebögen verwendeten 2 Teilnehmer. Informationsvideos und die digitale Unterschrift wurden von einem der Teilnehmer verwendet. Im Durchschnitt gaben die aufklärenden Ärzte an, dass ihrer Meinung nach der Text der Aufklärungsbogen von den Patienten nur zu 32 % gelesen wird. Keiner der aufklärenden Teilnehmer glaubte, dass die Patienten den Text vollständig verstehen. Im Mittel gaben die Teilnehmer an, dass die Patienten nach ihrer Meinung den Text zu 40,9 % verstehen. Bei den Anamnesefragen schätzen die Teilnehmer, dass die Patienten die Fragen zu 48,9 % verstehen.

Eine geschätzte Dauer des Aufklärungsgesprächs von unter 3 min gaben 26 Teilnehmer (27,9 %) an, eine Dauer von 3–5 min 50 Teilnehmer (53,8 %) und eine Dauer von 5–10 min 17 Teilnehmer (18,3 %). Der Mittelwert für die Aufklärungsdauer wurde von den Ärzten etwas niedriger geschätzt als von den Patienten (4,08 min vs. 4,7 min). Durch die CT-Aufklärungen fühlten sich 85,3 % der Teilnehmer stark oder sehr stark in ihrem Arbeitsablauf beeinträchtigt. Dies betraf 60 % (9 von 15) der Teilnehmer nichtradiologischer Fachrichtungen und 94,5 % der radiologisch tätigen Teilnehmer (67 von 74).

Die nächste Frage beschäftigte sich damit, wie ausführlich die Ärzte nach eigener Angabe hinsichtlich verschiedener Aspekte der Untersuchung aufklären. Die Ergebnisse sind in Tab. 3 dargestellt.

**Table 3:** 

	Anzahl der Teilnehmer, die über den entsprechenden Aspekt gar nicht, sehr wenig oder wenig aufklären (*n* = 90)	Anzahl der Teilnehmer, die über den entsprechenden Aspekt genau, ausführlich oder sehr ausführlich aufklären (*n* = 90)
Ablauf und Dauer der Untersuchung	46 (51,1 %)	44 (48,9 %)
Notwendigkeit der Untersuchung	41 (45,6 %)	49 (54,4 %)
Mögliche Alternativen	65 (72,2 %)	25 (27,8 %)
Strahlenrisiko	38 (42,2 %)	52 (57,8 %)
Nebenwirkung von Kontrastmitteln	6 (6,7 %)	84 (93,3 %)

**Figure 2:**
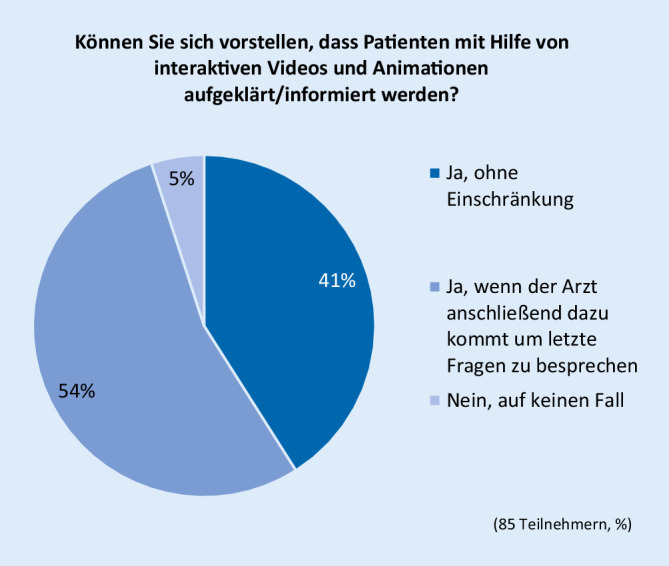

**Figure 3:**
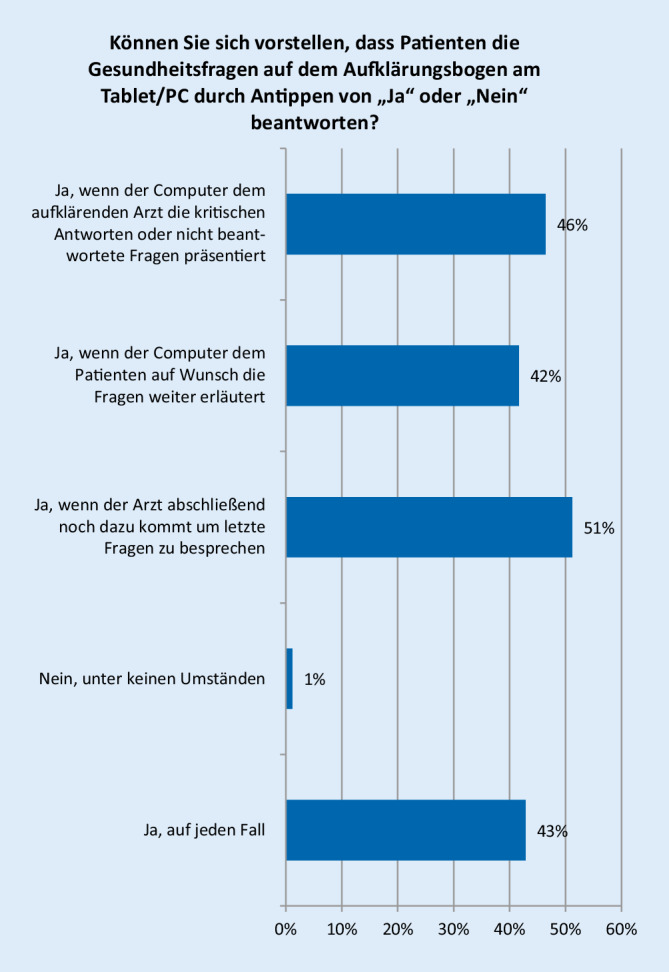

Der Großteil der Teilnehmer war technischen Neuerungen gegenüber positiv eingestellt. Die Fragen zur Einstellung gegenüber technischen Neuerungen beantworteten 85 Teilnehmer. Der Aufklärung mithilfe von interaktiven Videos und Animationen stimmten 95,3 % zu (Abb. 2). Fast alle Teilnehmer können sich vorstellen, dass Patienten die Anamnesefragen statt im Aufklärungsbogen am Tablet/PC beantworten (Abb. 3).

## Diskussion

### Aufklärungsinhalte aus Sicht der Patienten und der Ärzte

Im Rahmen einer Aufklärung, so auch für eine Computertomographie, soll der Patient nach § 630e BGB Absatz 1 über „Art, Umfang, Durchführung, zu erwartende Folgen und Risiken der Maßnahme sowie ihrer Notwendigkeit, Dringlichkeit, Eignung und Erfolgsaussichten im Hinblick auf die Diagnose“ in verständlicher und angemessener Weise informiert werden [3]. Bei der CT sind die Patienten neben dem Untersuchungsablauf und der Strahlenexposition auch über die teils seltenen Risiken, die bei der Verwendung von Kontrastmittel bestehen aufzuklären [7, 19]. Dazu gehören vor allem allergische Reaktionen, Nierenschädigungen oder Schilddrüsenfunktionsstörungen [11, 14, 23]. Diese wichtigen Punkte sollten folglich allen Patienten nach der Aufklärung bekannt sein. In der vorliegenden Befragung zeigte sich jedoch eine teilweise schlechte Erinnerungsfähigkeit der Patienten in Bezug auf die Aufklärungsinhalte.

So erinnerte sich beispielsweise nur etwas mehr als ein Drittel der Patienten an eine Aufklärung hinsichtlich einer möglichen Schilddrüsenfunktionsstörung oder nur knapp mehr als die Hälfte an eine Aufklärung über eine Nierenfunktionsstörung durch eine Kontrastmittelgabe. Die bei allen Kategorien etwas bessere Erinnerungsfähigkeit der Klinikpatienten im Vergleich zu den Teilnehmern der Online-Befragung ist sicherlich im engen zeitlichen Zusammenhang der Abfrage der erinnerten Inhalte mit dem eigentlichen Aufklärungsgespräch bei den Klinikpatienten zu sehen.

Betrachtet man die Merkfähigkeit hinsichtlich der Aufklärungsinhalte in den verschiedenen Altersgruppen, fällt eine mit dem Alter zunehmend schlechtere Merkfähigkeit auf. Lediglich im Hinblick auf den Aufklärungsinhalt „Strahlenbelastung/Strahlenrisiko“ zeigten sich keine wesentlichen Unterschiede zwischen den Altersgruppen.

In der vorliegenden Befragung klärten die meisten befragten Ärzte vor allem über die Nebenwirkungen von Kontrastmitteln auf. Über mögliche Alternativen und die Notwendigkeit der Untersuchung wurde weniger detailliert aufgeklärt, was sich auch in den Ergebnissen der Patientenbefragung widerspiegelte. An eine Information über alternative Untersuchungsmöglichkeiten erinnerten sich rund 92 % aller Patienten nicht. Die Aufklärung über Alternativen ist insbesondere bei onkologischen Patienten mit wiederkehrenden Schnittbildgebungen wichtig [10, 29]. Eine Ursache besteht möglicherweise darin, dass die rechtfertigende Indikation für die Durchführung der Untersuchung überwiegend bereits vor der Aufklärung gestellt wurde und die Abwägung gegenüber alternativen Methoden ebenfalls bereits erfolgt ist. Weiterhin zeigte die Befragung, dass sich zwischen 39,3 % (Klinik-Befragung) und 34,9 % (Online-Befragung) der Teilnehmer nicht an eine Aufklärung über die Strahlenbelastung erinnerten. In einer Befragung von Rodriguez et al. äußerten 73,5 % der Patienten den Wunsch nach einer Diskussion über das Strahlenrisiko [18]. Befragungen von Ärzten konnten in diesem Zusammenhang zeigen, dass die Einschätzung und das Bewusstsein bezüglich der Strahlenbelastung auch die aufklärenden Ärzte häufig vor eine Herausforderung stellt [9, 12, 30].

### Merkfähigkeit und Möglichkeiten zur Verbesserung

Als Merkfähigkeit bezeichnet man allgemein die Fähigkeit eines Menschen, sich an zuvor aufgenommene Informationen zu erinnern. Da der Mensch ein begrenztes Kurzeitgedächtnis besitzt, ist es nicht leicht, Informationen längerfristig zu behalten. Dieser allgemeine Grundsatz gilt auch für Aufklärungsgespräche für eine CT-Untersuchung. Während eines Gesprächs, so auch in einem Aufklärungsgespräch, muss der Zuhörer direkt entscheiden, welche Inhalte er versteht, als entscheidend ansieht und zu welchen Themen er gegebenenfalls durch gezielte Rückfragen genauere Informationen erhält. Durch eine solche Vertiefung und Wiederholung von Informationen kann sich die Merkfähigkeit verbessern. Zusätzlich können visuelle und akustische Reize dabei helfen, sich aufgenommene Informationen besser und länger zu merken.

Im medizinischen Kontext könnten PC- bzw. Tablet-basierte Aufklärungen eine Möglichkeit zur Verbesserung der Merkfähigkeit der Patienten hinsichtlich der Aufklärungsinhalte sein. So konnten Rowbothan et al. in ihrer Studie zur Aufklärung für eine Studienteilnahme eine bessere Merkfähigkeit der Patienten durch eine Tablet-basierte Aufklärung im Vergleich zu einer klassischen schriftlichen Variante erreichen [20]. Durch den unterstützenden Einsatz von PCs oder Tablets mit Videos in verschiedenen Sprachen und der Möglichkeit, sich Teile wiederholt anzusehen, könnte die Aufklärung der Patienten zudem individualisiert werden.

### Kritik der Patienten und Ärzte

Interessant ist, dass die aufklärenden Ärzte annahmen, dass die Texte der Aufklärungsbögen von den Patienten nur zu etwa einem Drittel gelesen werden und keiner der Teilnehmer glaubte, dass die Patienten den Text vollständig verstehen. Von den Patienten werden häufig die Sprache und die verwendeten Fachbegriffe kritisiert. Bei der Aufklärung und Information der Patienten sollte daher auf eine leicht verständliche Sprache ohne Fachbegriffe geachtet werden [8].

### Arbeitsbelastung der Ärzte durch Aufklärungen

Nach der vorliegenden Befragung klärte über ein Drittel der befragten Ärzte mehr als 10 Patienten pro Tag auf. Der Maximalwert lag bei 35 Patientenaufklärungen pro Tag. Die vorliegende Befragung verdeutlicht, dass die Ärzte durch die Anzahl und den Umfang der Aufklärung ein zeitliches/organisatorisches Problem haben. So hatten 94,5 % der radiologisch tätigen Teilnehmer angegeben, dass die Aufklärungen sie stark oder sehr stark in ihrem Arbeitsablauf beeinträchtigen. Dies liegt sicherlich teils auch an der zusätzlichen ausführlichen Dokumentation des Ausklärungsgesprächs. Schlechtweg et al. verglichen in ihrer Studie eine Tablet-basierte Aufklärung vor einer Magnetresonanztomographie (MRT) mit der klassischen schriftlichen Form [21, 22]. Neben dem Vorteil der digitalen Speicherung zeigte sich in der Tablet-basierten Gruppe zudem eine lückenlose Beantwortung der Gesundheitsfragen, wohingegen 11 % der schriftlichen Aufklärungen unvollständig waren. Um den mündlichen Teil des Aufklärungsgesprächs zu dokumentieren, sollen schriftliche Anmerkungen auf dem Aufklärungsbogen gemacht werden. Durch die Möglichkeit, diese über eine Tastatur am PC oder Tablet einzugeben, könnte neben einer vereinfachten Dokumentation zusätzlich das Problem der häufig schwer lesbaren ärztlichen Handschrift umgangen werden [6, 17, 24].

#### Delegation der Aufklärung

Nach § 630e Absatz 2 BGB muss eine Aufklärung „mündlich durch den Behandelnden oder eine Person erfolgen, die über die zur Durchführung der Maßnahme notwendige Ausbildung verfügt“ [3]. Die Delegation einer Aufklärung an nichtärztliches Personal ist nicht zulässig. Eine Delegation an einen anderen Arzt ist grundsätzlich möglich. Allerdings muss dieser, wie im wie in § 630e [2] formuliert, über die notwendige Qualifikation verfügen [5]. In vielen Krankenhäusern ist es Realität, dass auch fachfremde Ärzte die Patienten für eine CT-Untersuchung aufklären. Aus diesem Grund haben sich auch Nichtradiologen an dieser Befragung beteiligt. Auch wenn erfahrene Nichtradiologen die Risiken einer radiologischen Untersuchung sicherlich gut einschätzen und die Aufklärung gewissenhaft durchführen, ist diese aus juristischer Sicht nur begrenzt delegationsfähig. Den delegierenden Arzt, im Fall einer CT-Aufklärung also den Radiologen, treffen hierbei Kontroll- und Überwachungspflichten. So hat er sicherzustellen, dass der betraute Arzt seiner Pflicht ordnungsgemäß nachkommt, um haftungs- und strafrechtliche Folgen zu vermeiden [5].

### Akzeptanz technischer Neuerungen

Der letzte Teil der Befragung beschäftigte sich mit der Akzeptanz technischer Neuerungen bei der Aufklärung. Hier zeigte sich über alle Altersstufen eine große Bereitschaft, z. B. mithilfe von Videos und Animationen über die Computertomographie aufgeklärt/informiert zu werden. Ein sehr großer Teil, insgesamt 88,7 % der Teilnehmer, würde diese Möglichkeit nutzen. Immerhin 74 % der Teilnehmer würden die Gesundheitsfragen am Tablet oder PC beantworten. Besonders deutlich ist das Ergebnis der Befragung unter den Ärzten. 95,3 % der ärztlichen Teilnehmer würden die Aufklärung von Patienten mithilfe von interaktiven Videos und Animationen und sogar 98,8 % die Beantwortung der Gesundheitsfragen am Tablet oder PC befürworten.

Ein möglicher Ansatzpunkt zur Verbesserung der in dieser Studie genannten Probleme ist in der Aufklärung mit Hilfe von Videos zu sehen, die verständlich und anschaulich entsprechend der Erwartungen/Bedürfnisse der Patienten gestaltet werden können. Der Einsatz solcher Informationsvideos zusammen mit Tablet‑/PC-gestützten Aufklärungsbögen könnten neben einer Vereinfachung der Dokumentation auch zu einer besseren Information der Patienten und einer Entlastung der aufklärenden Ärzte führen.

## Fazit

- Die Erinnerungen der befragten Patienten an die Inhalte des Aufklärungsgesprächs waren teilweise nur gering.
- Die Dauer des Aufklärungsgesprächs schätzten die befragten Ärzte im Vergleich zu den Patienten etwas länger ein.
- Die meisten befragten Ärzte klärten vor allem über die Nebenwirkungen von Kontrastmitteln auf; über die Notwendigkeit der Untersuchung und mögliche Alternativen wurde weniger aufgeklärt.
- Die Aufklärung mittels Informationsvideos und die Beantwortung der Gesundheitsfragen mittels Tablet/PC wurden von einem Großteil der teilnehmenden Patienten und Ärzte befürwortet.
